# CCR9 interactions support ovarian cancer cell survival and resistance to cisplatin-induced apoptosis in a PI3K-dependent and FAK-independent fashion

**DOI:** 10.1186/1757-2215-3-15

**Published:** 2010-06-17

**Authors:** Erica L Johnson, Rajesh Singh, Crystal M Johnson-Holiday, William E Grizzle, Edward E Partridge, James W Lillard, Shailesh Singh

**Affiliations:** 1 Department of Microbiology, Biochemistry and Immunology, Morehouse School of Medicine, 720 Westview Drive SW, Atlanta, GA 30310, USA; 2 Department of Pathology, University of Alabama at Birmingham, 619 South 19th Street, Birmingham, AL 35233, USA

## Abstract

**Background:**

Cisplatin is more often used to treat ovarian cancer (OvCa), which provides modest survival advantage primarily due to chemo-resistance and up regulated anti-apoptotic machineries in OvCa cells. Therefore, targeting the mechanisms responsible for cisplatin resistance in OvCa cell may improve therapeutic outcomes. We have shown that ovarian cancer cells express CC chemokine receptor-9 (CCR9). Others have also shown that CCL25, the only natural ligand for CCR9, up regulates anti-apoptotic proteins in immature T lymphocytes. Hence, it is plausible that CCR9-mediated cell signals might be involved in OvCa cell survival and inhibition of cisplatin-induced apoptosis. In this study, we investigated the potential role and molecular mechanisms of CCR9-mediated inhibition of cisplatin-induced apoptosis in OvCa cells.

**Methods:**

Cell proliferation, vibrant apoptosis, and TUNEL assays were performed with or without cisplatin treatment in presence or absence of CCL25 to determine the role of the CCR9-CCL25 axis in cisplatin resistance. In situ Fast Activated cell-based ELISA (FACE) assays were performed to determine anti-apoptotic signaling molecules responsible for CCL25-CCR9 mediated survival.

**Results:**

Our results show interactions between CCR9 and CCL25 increased anti-apoptotic signaling cascades in OvCa cells, which rescued cells from cisplatin-induced cell death. Specifically, CCL25-CCR9 interactions mediated Akt, activation as well as GSK-3β and FKHR phosphorylation in a PI3K-dependent and FAK-independent fashion.

**Conclusions:**

Our results suggest the CCR9-CCL25 axis plays an important role in reducing cisplatin-induced apoptosis of OvCa cells.

## Background

OvCa is the most lethal among gynaecologic malignancies [1]. Cancer cells develop resistance to chemotherapy by inactivating apoptotic factors and enhancing survival pathways that antagonize apoptotic signals [2]. The first-line chemotherapeutic agent for OvCa is cisplatin. Unfortunately, many ovarian tumors show resistance to cisplatin, characterized by decreased susceptibility to apoptosis. Intracellular signalling by chemokine receptors primarily involves Gαi, along with the Gβ-Gγ dimer of the heterotrimeric G proteins [3], which activate the PI3K/Akt pathway. Downstream mediators of PI3K directly induce Akt activation [4]. Phosphorylated Akt promotes cell survival by inactivating pro-apoptotic factors, such as forkhead transcriptional factor (FKHR) and glycogen synthase kinase-3β (GSK-3β) [5]. Hence, this anti-apoptotic-survival pathway has been shown to play a significant role in cisplatin resistance [6].

CCR9 signalling has been shown to facilitate immature T cell survival through PI3K and Gαi protein-dependent activation of Akt [7]. Alternatively, chemokine receptors aggregate with associated integrins on lipid rafts following stimulation to promote FAK phosphorylation, which could presumably support anti-apoptotic mechanisms via FAK-Akt signaling. This study investigates the role of CCR9 signalling on OvCa cell survival and cisplatin resistance. We show for the first time that CCL25-CCR9 interactions in OvCa cells provide protection against cisplatin-induced cell death. We also report that CCL25 promotes proliferation and CCR9-dependent anti-apoptotic signalling via the PI3K/Akt/GSK/FKHR pathway and independent of FAK. These studies suggest expression of functional CCR9 contributes to ovarian tumor cell survival.

## Methods

### Cell Lines and cell culture

Human OvCa cell line, OVCAR-3, was obtained from the ATCC. The cells were cultured in RPMI 1640 (Mediatech, Inc.) at 37°C and 5% CO_2 _with 10% fetal bovine serum (FBS; Sigma). The SKOV-3 cell line was obtained from Dr. Negrin [8]. SKOV-3 cells were cultured in Ham's F12K medium with 2 mM L-glutamine and adjusted to contain 1.5 g/L sodium bicarbonate (ATCC) with 10% FBS at 37°C with 5% CO_2_. After five passages in Ham's F12K media, SKOV-3 cells were switched to RPMI-1640 with 10% FBS. Prior to each experiment, cells were cultured for 24 hours in RPMI 1640 and 2% charcoal-striped FBS.

### Cell Proliferation Assay

OvCa cells (10^5^) were cultured alone or with 100 ng/ml CCL25 + 1 μg/ml of isotype control antibody or 100 ng/ml CCL25 + 1 μg/ml anti-CCR9 antibody (clone 112509, R&D Systems) for 24 hours with 0, 0.5, 5, 10, 25 and 50 μg/ml of cisplatin. Incorporation of bromodeoxyuridine (BrdU) into newly synthesized DNA permits indirect detection of rapidly proliferating cells. Hence, this assay was used according to manufacturer's instructions to estimate OvCa cell growth. Briefly, cells were treated with BrdU for 18 hours at 37°C. Media containing labelling solution was removed and cells were washed twice with media containing 10% serum. OvCa cells were fixed with 200 μl of fixative solution for 30 minutes at ~25°C and washed as before. Next, cells were incubated with 100 μl of nuclease solution for 30 minutes at 37°C and washed 3 times. Subsequently, 100 μl of anti-BrdU antibody was added, incubated for 30 minutes at 37°C, and washed 3 times. BrdU incorporation by OvCa cells was detected by peroxidase substrate reaction. After the extinction of this reaction, the samples were measured in a micro plate reader at 405 nm with a reference wavelength at approximately 490 nm.

### Vybrant Apoptosis Assay

OvCa cells were cultured with 0 or 5 μg/ml of cisplatin, along with no additions or 100 ng/ml of CCL25 plus 1 μg/ml of anti-CCR9 or isotype control antibodies for 24 hours. The cells were harvested and washed in cold PBS and the cell density was adjusted to 10^6 ^cells/ml. Subsequently, cells were stained with Annexin V and Propidium Iodide (PI) using the Vybrant #3 assay (Invitrogen), according to manufacturer's instructions. The stained cells were analyzed by flow cytometry using UV/488 nm dual excitation and the fluorescence emission was measured at 530 nm and 575 nm.

### Terminal Transferase dUTP Nick End Labeling (TUNEL) Assay

OvCa cells were cultured with 0 or 5 μg/ml of cisplatin, along with no additions or 100 ng/ml of CCL25 plus 1 μg/ml of anti-CCR9 or isotype control antibodies for 24 hours. Apoptosis was measured by TUNEL assay (Millipore) according to the manufacturer's instructions. Briefly, following treatment the cells were fixed with 4% paraformaldehyde in 0.1 M NaH_2_PO_4_, 7.4 pH for 15 minutes. After washing in PBS three times, the cells were incubated with 0.05% Tween-20 in PBS for 15 minutes. After washing in PBS, the cells were incubated with TdT end-labelling cocktail for 60 minutes. Termination buffer was added to stop the reaction. After washing 4 times in PBS, cells were blocked for 20 minutes and stained with avidin-fluorescein isothiocyanate (FITC) solution for 30 minutes. After washing with PBS 3 times, fluorescence plate reader quantified the fluorescence of TUNEL positive cells.

### Fast Activated cell-based ELISA (FACE) assay

The level of total and phosphorylated PI3Kp85-Tyr, Akt-Ser473, GSK-3β-Ser9, and FKHR-Thr24 were quantified using Fast Activated Cell-based ELISA (FACE) assays (Active Motif) according to the manufacturer's protocol. Briefly, OvCa cells were cultured in 96-well plates (5 × 10^3 ^cells/well) 200 μl of culture medium (in triplicate for each treatment) one day prior to manipulation. OvCa cells were treated with 0 or 5 μg/ml of cisplatin, along with no additions or 100 ng/ml of CCL25 plus 1 μg/ml of anti-CCR9 or isotype control antibodies for 24 hours. In addition, cells were treated with or without kinase inhibitors of PI3K (wortmannin, Sigma), and FAK (PF-573, 228, Pfizer). Cells were then fixed with 4% formaldehyde at room temperature for 20 minutes, followed by washing with PBS containing 0.1% Triton X-100. Endogenous peroxidase activity was quenched using 1% H_2_O_2 _in wash buffer. The cells were incubated in antibody blocking buffer, followed by incubations with phospho- or total anti-PI3Kp85-, or Akt- or GSK-3β, -FKHR-specific primary antibodies. After washing steps, horse raddish peroxidase (HRP)-conjugated antibody was added and cells were incubated for one hour at ~25°C. Subsequently, the plates were developed and chemiluminescence was measured using a Spectramax-2 plate reader (Molecular Devices). Finally, plates were washed and the number of cells in each well was estimated by crystal violet staining, measuring absorbance at 595 nm. Relative cell numbers were then used to normalize chemiluminescent readings, and the change in phosphorylation status was calculated by dividing chemiluminscence detected using phospho protein-specific antibody with that of the total protein-specific antibody.

### Statistics

The data were compared using a two-tailed Student's t test and expressed as the mean ± SE. The results were analyzed using the Stat view II program (Abacus Concepts, Inc.) and were labelled statistically significant if *p *values were < 0.01.

## Results

### Effects of CCL25 on cisplatin-induced growth inhibition

**Figure 1:**
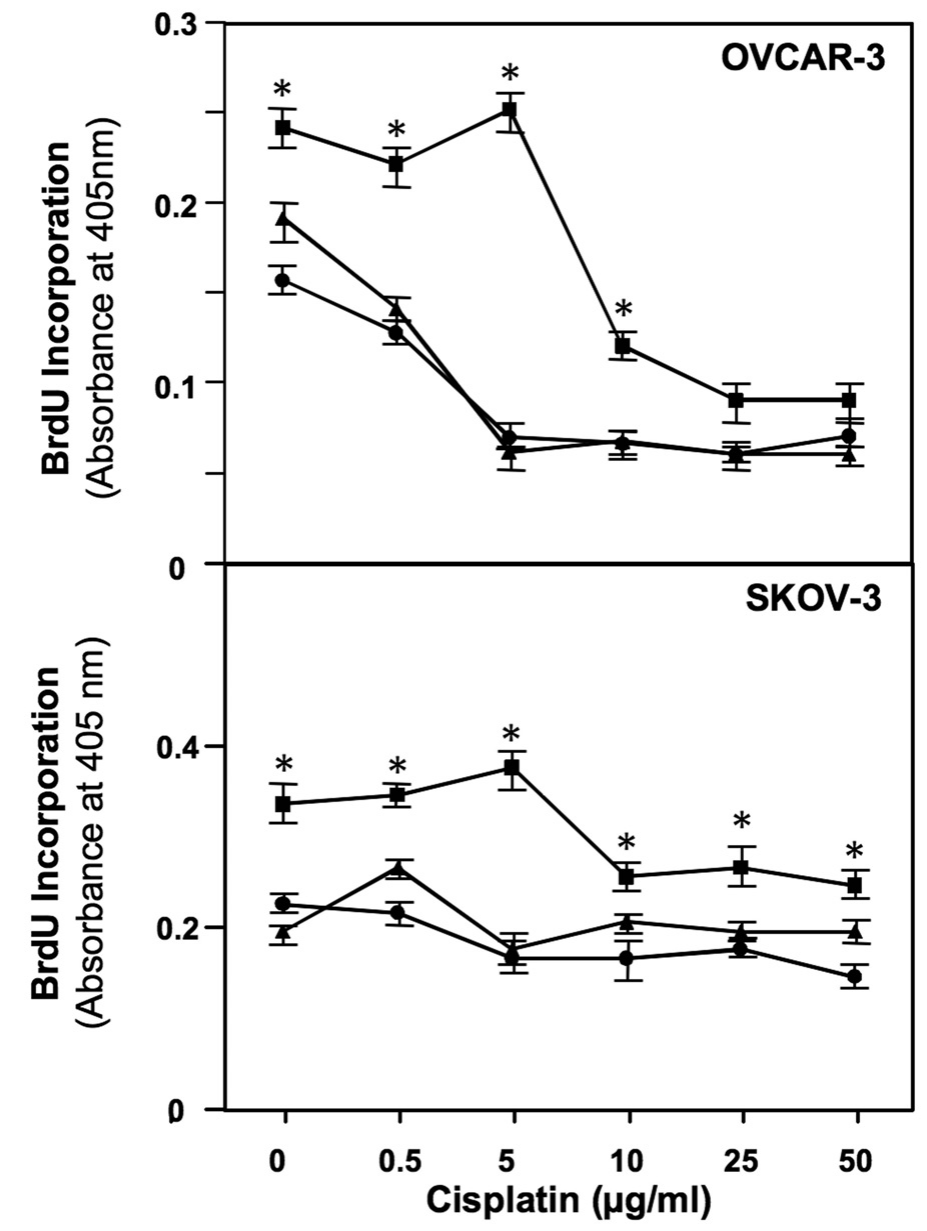
**CCL25 inhibits cisplatin-induced cell death**. OVCAR-3 and SKOV-3 cells were cultured with 0 (circles) or 100 ng/ml of CCL25 plus isotype control (squares) or anti-CCR9 (triangles) antibodies for 24 hours, along with increasing concentrations of cisplatin. Cell proliferation was determined by BrdU incorporation and assays were repeated 3 times and performed in triplicate. Asterisk(s) (*) indicate statistical significant differences (*p *< 0.01) between CCL25-treated and untreated OvCa cells.

SKOV-3 cells incorporated BrdU at a higher rate than OVCAR-3 cells (0.375 *versus *0.250 OD_405 nm_, respectively), which suggested SKOV-3 cells proliferated at a higher rate compared to OVCAR-3 cells (Figure 1). In the absence of cisplatin, CCL25 significantly enhanced BrdU incorporation (i.e., growth) of OVCAR-3 and SKOV-3 cell lines by ~ 1.5-fold in comparison to untreated cells. However, when these cells were treated with increasing concentrations of cisplatin, CCL25 protected human OvCa cells from cisplatin-mediated growth inhibition. CCL25 optimally protected against 5 μg/ml or less cisplatin with 3.5 and 2.2-fold increases in OVCAR-3 and SKOV-3 cell BrdU incorporation respectively, in comparison to the untreated cells or CCL25 plus anti-CCR9 antibody treated cultures. In general, CCL25 treatment abrogated the growth inhibition of OVCAR-3 and SKOV-3 cell lines caused by cisplatin in a CCR9-dependent fashion.

### CCL25-induced cisplatin resistance of OvCa cell lines

**Figure 2:**
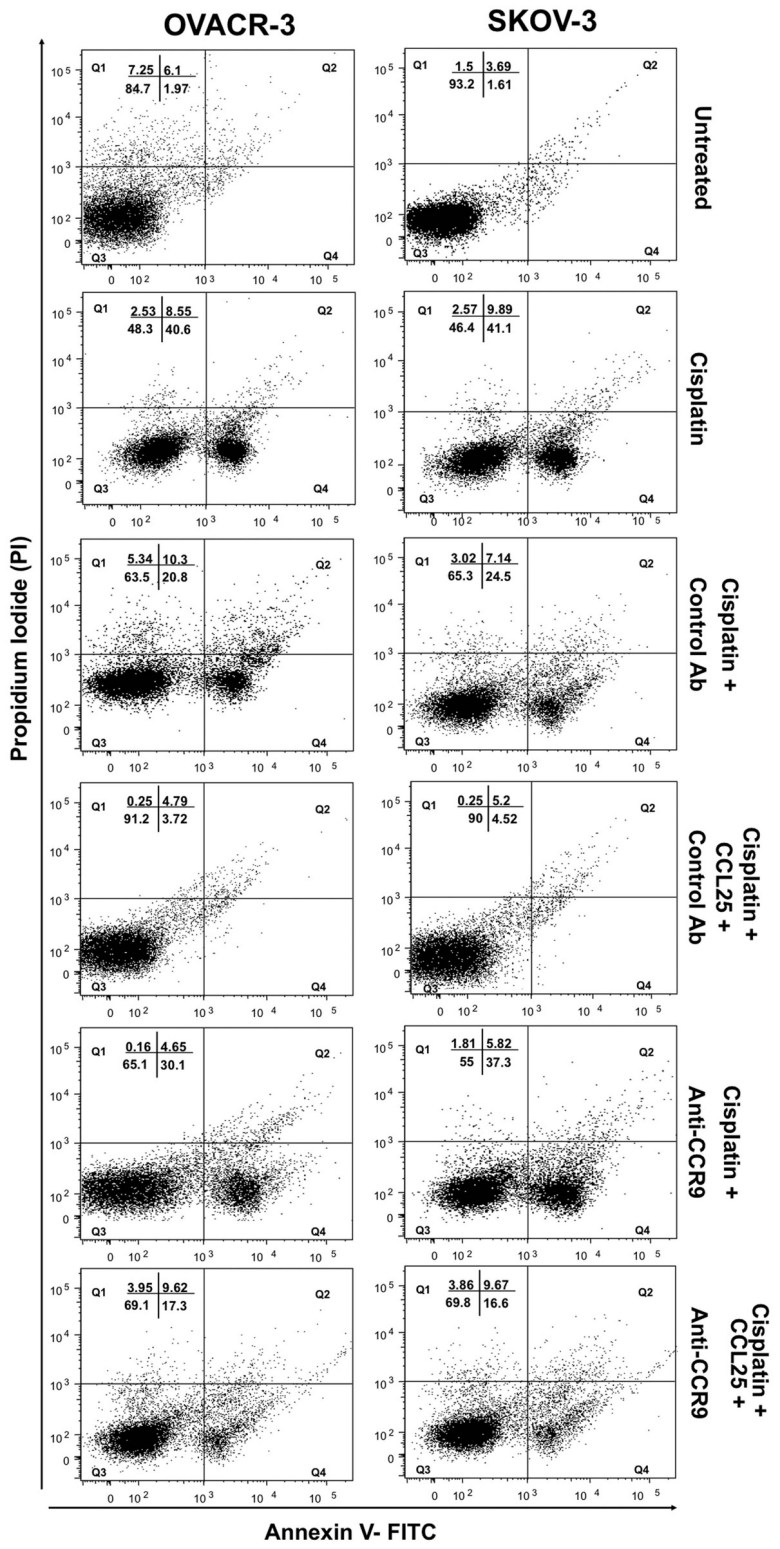
**Percent change in the number of cisplatin-induced apoptotic OvCa cells**. OVCAR-3 and SKOV-3 cells were cultured for 24 hours with 5 μg/ml of cisplatin alone or with 0 or 100 ng/ml CCL25 plus 1 μg/ml of anti-human CCR9 or isotype and untreated cell were used as controls. Cells were harvested and stained with annexin V and propidium iodide. Dual flowcytometric analysis of Annexin V-FITC and propidium iodide (PI) staining. Living cell populations are clustered in the Q3 quadrant; cells in early apoptosis are in the Q4 quadrant; late apoptotic/necrotic cells are in the Q2 quadrant.

**Figure 3:**
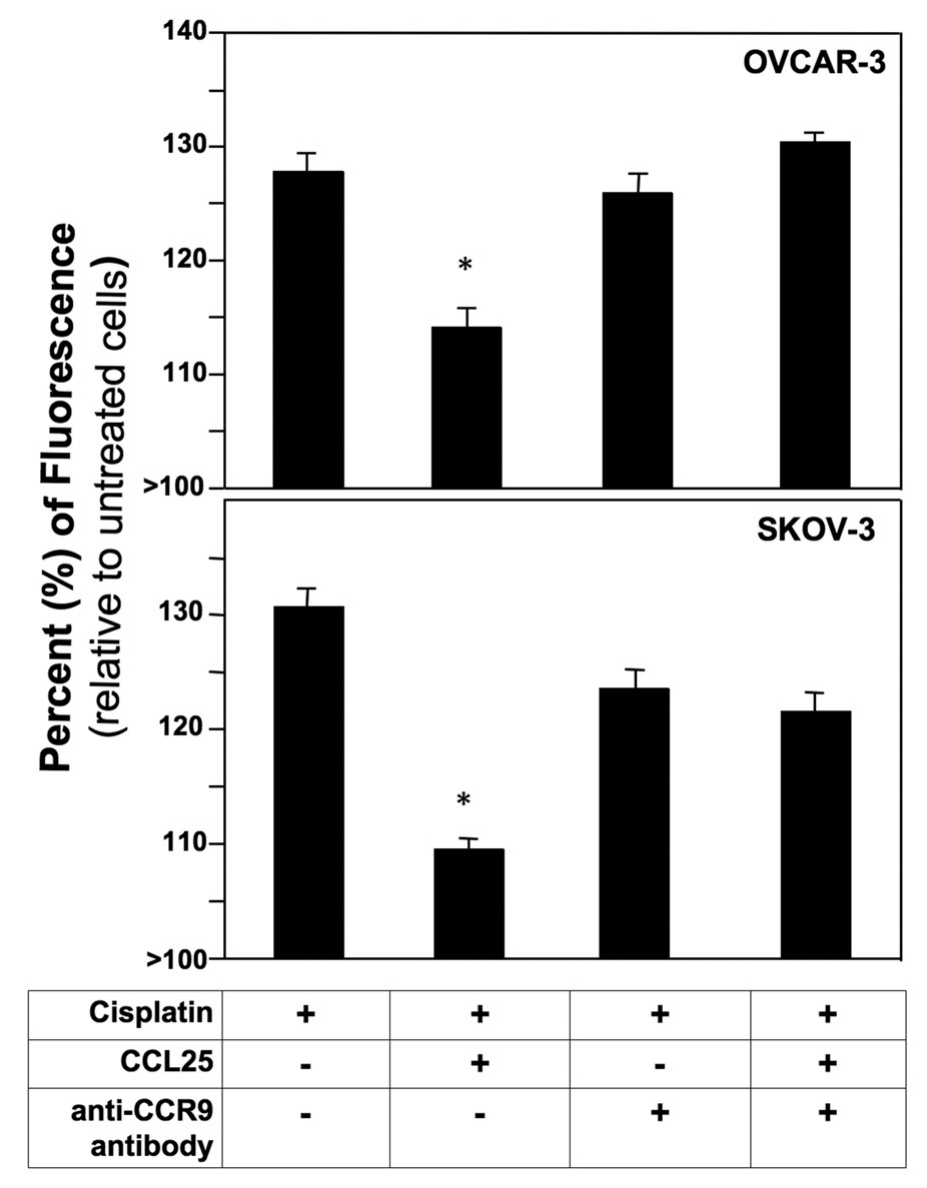
**Percent change in the number of cisplatin-induced Tunel-positive OvCa cells**. OVCAR-3 and SKOV-3 cells were cultured for 24 hours with 5 μg/ml cisplatin or with 0 or 100 ng/ml of CCL25 plus 1 μg/ml of anti-human CCR9 or isotype control antibodies. Detection of apoptotic cells was carried out using the terminal deoxynucleotidyl transferase-mediated dUTP nick-end labelling (TUNEL) method. Apoptotic cells exhibited nuclear green fluorescence with a standard fluorescence filter set (520 ± 20 nm). Asterisks (*) indicate statistical significant differences (*p *< 0.01) between treated and untreated OvCa cells.

Treatment of OVCAR-3 and SKOV-3 cell lines with cisplatin alone resulted in 96% and 95% respective increases in apoptosis relative to the untreated cells (Figure 2). CCL25 treatment significantly lowered the percentage of apoptotic OVCAR-3 and SKOV-3 cells. However, when the OvCa cell lines were treated with anti-CCR9 antibody or CCL25 + anti-CCR9 antibody, the percentage of apoptotic cells was restored to levels of observed with cisplatin treatment alone. Apoptosis was also assessed under the same conditions by TUNEL assay. OvCa cell lines treated with cisplatin alone resulted in ~130% increase in apoptosis relative to the untreated cells (Figure 3). The percentage of apoptotic cells was significantly lower than controls when cells were treated with cisplatin and CCL25. However, this CCL25-mediated survival was significantly reduced by CCR9 blockade.

### CCL25-CCR9 interactions impact on PI3Kp85-phospho Tyr and Akt-Ser473 activation

**Figure 4:**
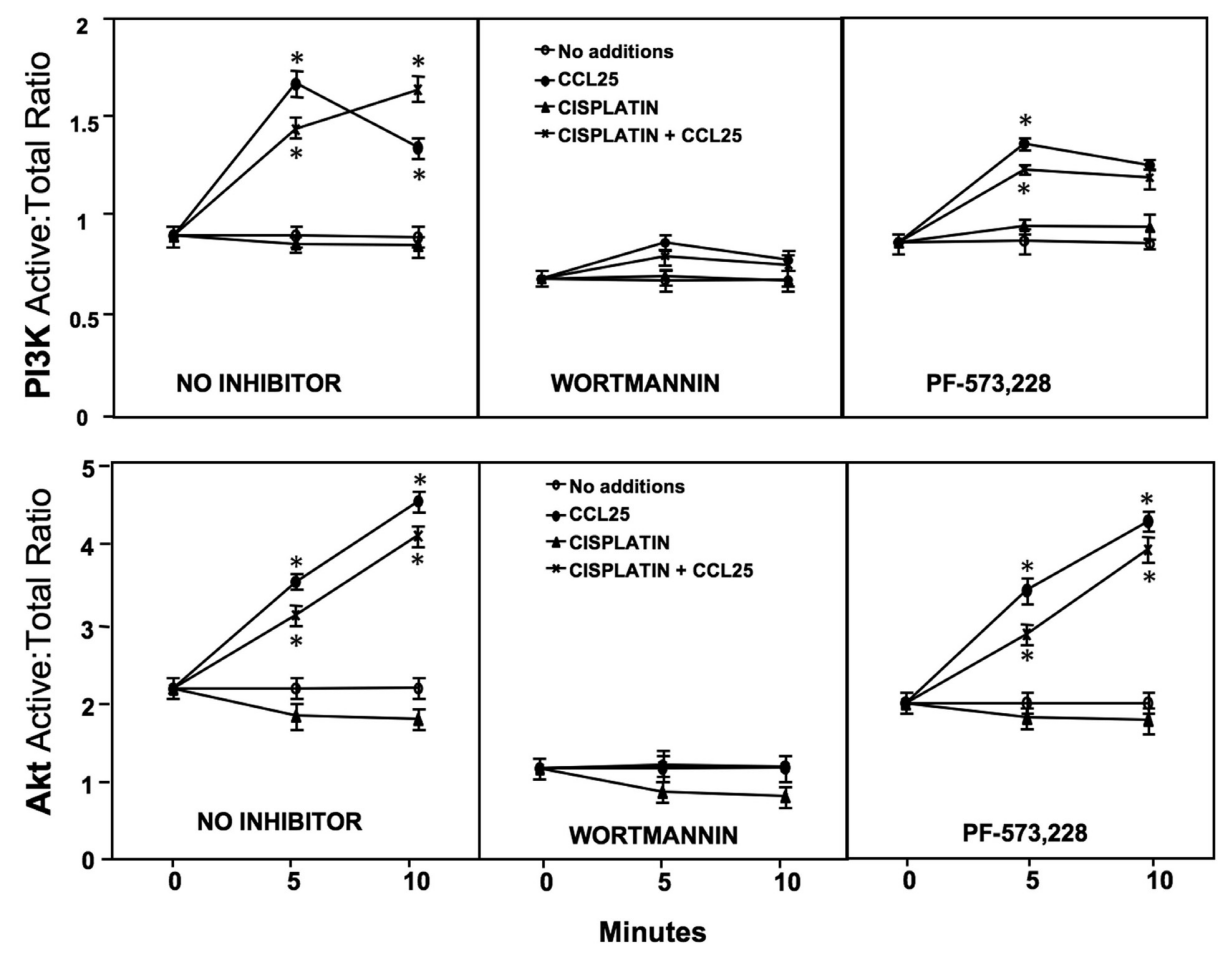
**PI3K and Akt activation after CCL25 treatment**. Cells were tested for their ability to phosphorylate PI3Kp85-tyrosines and Akt-Ser473 following treatment with or without CCL25, cisplatin, wortmannin, and/or PF-573,228. FACE assays quantified *in situ *total and phosphorylated protein levels before (0) or after (5 or 10 minutes) CCL25 stimulation in the presence of cisplatin and inhibitors. The ratio of active (phosphorylated) to total PI3K or Akt are presented ± SEM from 3 separate experiments performed in triplicate. Asterisks (*) indicated statistical differences (*p *< 0.01) between untreated and treated cells.

To determine the CCR9-mediated signals involved in OvCa cell survival, we performed FACE assays for PI3Kp85 phosphorylation (Figure 4). CCL25 induced a significant increase in PI3Kp85 activation within 5 minutes. This increase was lower after 10 minutes, but was still significantly higher than levels displayed by untreated cells (i.e., 0 ng/ml of CCL25). As expected, the PI3K inhibitor, wortmannin, reduced this increase. However, CCL25-treated OvCa cells co-incubated with the FAK inhibitor, PF-573, 228, continued to activate PI3Kp85. When treated with cisplatin alone, there was no difference in PI3Kp85 activity in comparison to the untreated cells. However, PI3Kp85 phosphorylation during cisplatin treatment significantly increased 10 minutes post CCL25 co-incubation. Similarly, cisplatin treatment had no effect on PI3Kp85 phosphorylation in the FAK-inhibited cells, while the cisplatin and CCL25 combination induced an immediate rise in PI3K activation, followed by a slight decrease. CCL25 also induced a gradual increase in Akt phosphorylation 5 and 10 minutes after treatment. Wortmannin treatment abrogated this increase, but CCL25-treated OvCa cells co-incubated with the FAK inhibitor continued to activate Akt at significantly high levels. Cisplatin treatment did not affect Akt phosphorylation, but CCL25 plus cisplatin treatment caused significant increases in Akt phosphorylation. While wortmannin pretreatment inhibited this CCL25-mediated Akt activity, cisplatin plus FAK inhibitor-treated and CCL25 co-incubated cells had the same level of enhance Akt phosphorylation:total protein levels as OvCa cells treated with CCL25 alone.

### GSK-3β-Ser9 and FKHR-Thr24 phosphorylation following CCL25-CCR9 interactions

**Figure 5:**
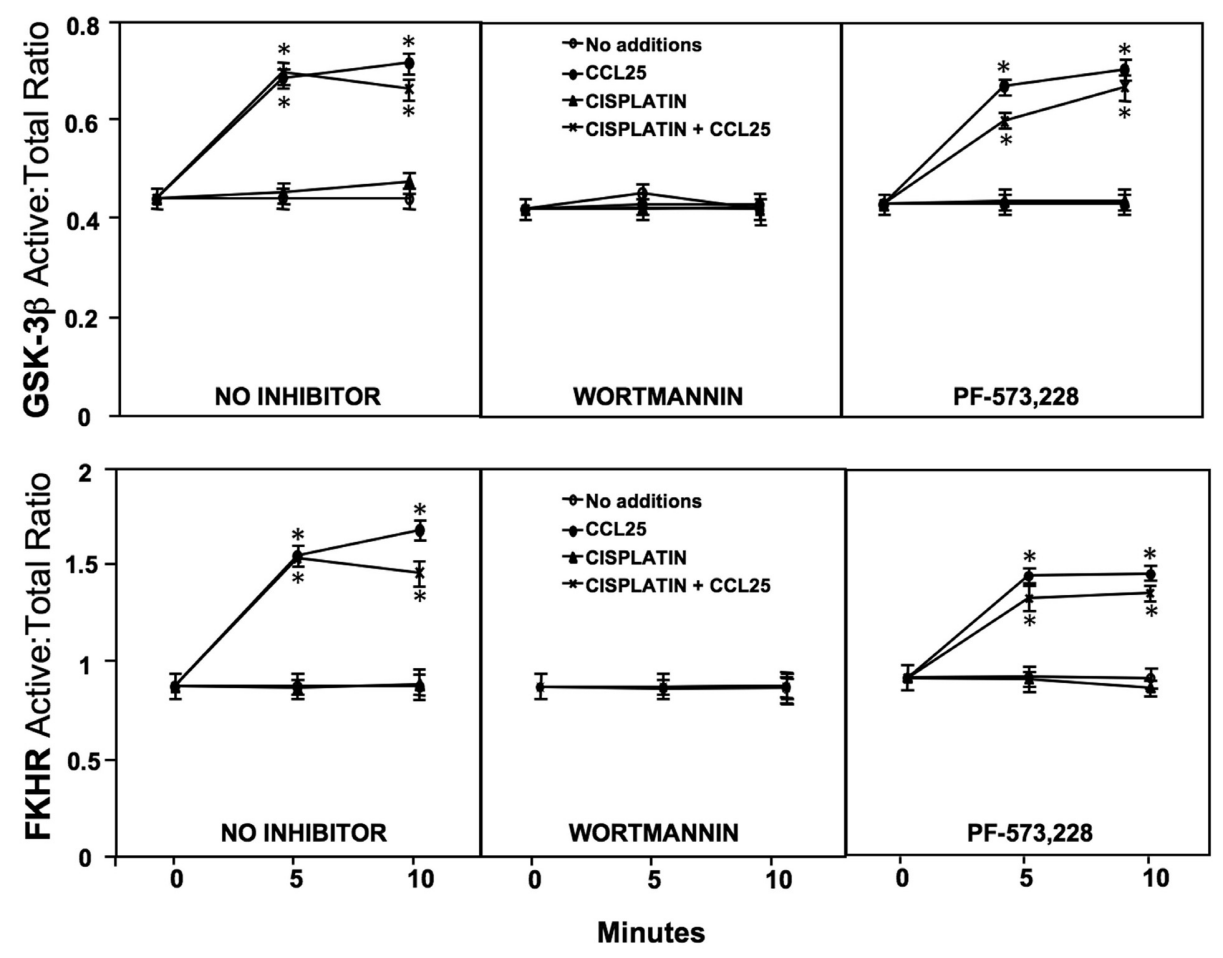
**GSK-3β and FKHR phosphorylation following CCL25 treatment**. Cells were tested for their ability to phosphorylate GSK-3β-Ser9 and FKHR-Thr24 following treatment with or without CCL25, cisplatin, wortmannin, and/or PF-573, 228. FACE assays quantified *in situ *total and phosphorylated protein levels before (0) or after (5 or 10 minutes) CCL25 stimulation in the presence of cisplatin and inhibitors. The ratio of phosphorylated:total GSK-3β or FKHR are presented ± SEM from 3 separate experiments performed in triplicate. Asterisks (*) indicated statistical differences (*p *< 0.01) between untreated and treated cells.

Akt inactivates GSK-3β and FKHR through phosphorylation. Hence, GSK-3β and FKHR FACE phosphorylation assays were performed to determine the effect of CCL25 on these regulators of cell survival. CCL25 treatment significantly increased phosphorylated:total GSK-3β protein levels after 5 and 10 minutes treatments or combined with cisplatin, compared to untreated cells (Figure 5). Wortmannin treatment completely abolished CCL25-mediated GSK-3β phosphorylation. Interestingly, PF-573, 228 plus CCL25 treatment had no effect on phosphorylation:total GSK-3β protein levels. Moreover, cisplatin treatment had no effect on CCL25-mediated GSK-3β phosphorylation, since CCL25 treatment of OvCa cells co-incubated with cisplatin significantly increased phosphorylated:total GSK-3β protein levels after 5 and 10 minutes treatments. This activation was inhibited by wortmannin treatment, but not by the FAK inhibitor. CCL25 also significantly increased phosphorylated:total FKHR levels after 5 and 10 minutes treatments, compared to untreated cells. Wortmannin, but not PF-573, 228, treatment inhibited this increase. Neither cisplatin treatment alone or in combination with the FAK inhibitor affected FKHR phosphorylation following CCL25 treatment. However, wortmannin treatment completely abrogated the CCL25-mediated increases in FKHR phosphorylation in cisplatin-treated OvCa cells.

## Discussion

A major cause of the high mortality rates due to OvCa is chemotherapy resistance. Cisplatin is often the first drug of choice for OvCa treatment. Unfortunately, cisplatin resistance is a major obstacle that impedes successful chemotherapy and a major cause of treatment failure in human OvCa. The balance between survival and apoptotic signals determine a cell's sensitivity to chemotherapy. Indeed, cancer cells develop resistance to chemotherapy by means of inactivating apoptotic factors and enhancing survival pathways that antagonize apoptosis signals [2]. However, the precise mechanisms of OvCa cell cisplatin-sensitivity or survival are not known.

Chemokines function to direct leukocyte and cancer cell migration, and play pivotal roles in cell survival [9]. Studies have demonstrated that CXCR4-CXCL12 interactions promote the survival of tumor cells, allowing growth under less favourable conditions. In particular, CXCR4 mediates survival in glioma cells [10]. Recent studies have also suggested that CCR9-CCL25 interactions potentiate anti-apoptotic signalling to immature T cells [11]. We have demonstrated that OvCa cells and tissues express CCR9 and play important role in cell migration, invasion under the chemotactic gradient of CCL25 (unpublished observations). Here we show that CCR9 also supports OvCa cell survival and cisplatin resistance. For the first time, we show that CCL25 significantly increases the proliferation of the OvCa cells in a CCR9-dependent fashion. In the presence of cisplatin, CCL25 also supported OvCa cell survival. Even though higher doses of cisplatin abrogated this CCL25-mediated resistance, our findings demonstrate that CCL25 confers significant cisplatin resistance.

Studies have shown that CCR9 signalling plays a role in immature T cell survival through PI3K and Gα_i _protein-dependent activation of Akt/protein kinase B [11]. By phosphorylation of its downstream effectors, Akt propagates cell survival signalling that promotes cell proliferation, maintains cell growth and inhibits apoptosis. The PI3K/Akt pathway has also been shown to be involved in cisplatin resistance. Recent studies show that Akt inactivation, through a PI3K inhibition, sensitizes OvCa cells to cisplatin-induced cell death [6]. Phosphorylated Akt promotes survival by phosphorylating and inactivating pro-apoptotic factors, such as FKHR and GSK-3β [7]. FKHR is a transcription factor that transactivates the expression of death-activating proteins, such as Fas ligand (FasL) and Bim [12]. Phosphorylation of FKHRL1 at Thr32, Ser253 and Ser315 prevents translocation of this protein to the nucleus and loss of FKHR-mediated gene transcription [13]. Recently, it was shown that activation of chemokine receptors lead to phosphorylation of GSK-3β and FKHR in a PI3K/Akt-dependent manner [14].

Taken together, our studies strongly support that CCR9-CCL25 signalling enhances OvCa survival and cisplatin resistance. Specifically, we show that CCL25 induces robust activation predominately through the PI3K/Akt pathway and its downstream mediators, FKHR and GSK-3β. Moreover, PI3K inhibition completely abrogated CCL25-mediated and CCR9-dependent cisplatin resistance, Akt, GSK-3β, and FKHR phosphorylation.

Chemokines-chemokine receptor interactions also support integrin clustering, which also increase FAK activation. FAK is a cytoplasmic protein tyrosine kinase involved in the regulation of cell proliferation, migration, and survival. FAK is constitutively associated with β-integrins. Activated FAK has also been shown to support PI3Kp85 phosphorylation following integrin clustering, but the mechanism(s) is not fully understood [15]. FAK inhibition did not effect CCL25-mediated PI3K, Akt, FKHR, or GSK-3β phosphorylation in OvCa cells, which suggest CCR9 signalling and survival mechanisms are independent of FAK activity.

**Figure 6:**
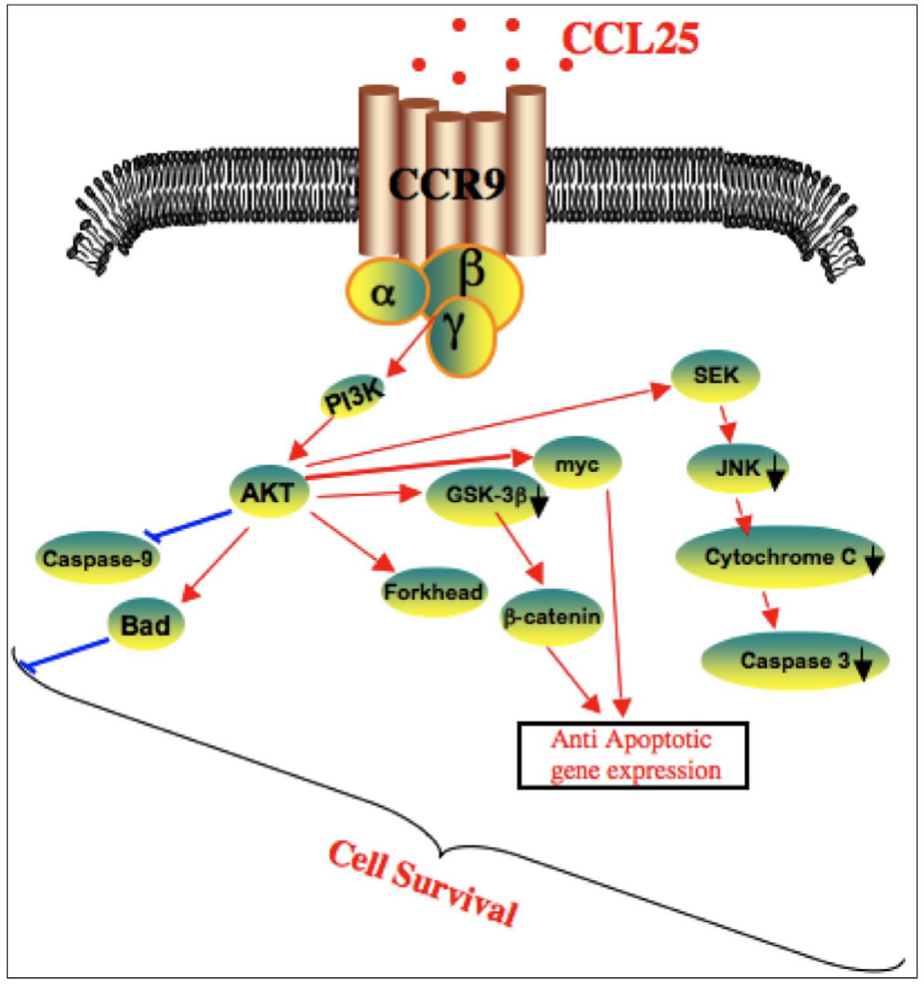
**Inhibition of apoptotic signal in cisplatin resistant tumor cells**. More than one mechanism is usually observed in resistant cells, and this contributes to the multi-factorial nature of cisplatin resistance. CCR9-dependent anti-apoptotic signalling in OvCa cells involves the PI3K/Akt cascade and phosphorylation of its downstream mediators, GSK-3β and FKHR.

Conflicting studies demonstrated cisplatin activates Akt in several cancer cell lines, which leads to cisplatin resistance [16]. Moreover, it has been shown that cisplatin can transiently induce Akt-mediated phosphorylation of FKHRL1 in the cisplatin-resistant cell line, CAOV-3, with subsequent cytoplasmic retention of FKHRL1 and cell survival [17]. However, cisplatin-treatment alone did not lead to significant increases in phosphorylation of PI3K, Akt, GSK-3β, or FKHR. In fact, cisplatin treatment led to a slight down regulation of Akt activation. However in the presence of CCL25 along with cisplatin, phosphorylation of Akt, GSK-3β and FKHR elevated to significant levels. Taken together, these results suggest that CCL25 treatment contributes to OvCa survival and cisplatin resistance. Moreover, we show that CCR9-dependent anti-apoptotic signalling in OvCa cells involves the PI3K/Akt cascade and phosphorylation of its downstream mediators, GSK-3β and FKHR (Figure 6).

## Conclusions

Our results suggest that rapid activation of the PI3K/Akt pathway occurs directly through the chemokine receptor/G-proteins and independent of FAK activation. These results support our hypothesis that CCL25-CCR9 interaction promotes OvCa survival and resistance to cisplatin.

## Competing interests

The authors declare that they have no competing interests.

## Authors' contributions

ELJ conducted the experiments, analyzed data, and drafted the manuscript. RS and CJH assisted with experiments and manuscript preparation. WEG, EEP, JWL and SS conceptualized, edited, and/or revised the manuscript. All authors have read and approved the final manuscript.
